# Parent-Child Agreement on Parent-to-Child Maltreatment

**DOI:** 10.1007/s10896-016-9902-3

**Published:** 2016-12-15

**Authors:** Laura H.C.G. Compier-de Block, Lenneke R.A. Alink, Mariëlle Linting, Lisa J.M. van den Berg, Bernet M. Elzinga, Alexandra Voorthuis, Marieke S. Tollenaar, Marian J. Bakermans-Kranenburg

**Affiliations:** 1 Centre for Child and Family Studies, Leiden University, Leiden, The Netherlands; 2 Faculty of Law, VU University Amsterdam, Amsterdam, The Netherlands; 3 Clinical Psychology Unit, Leiden University, Leiden, The Netherlands

**Keywords:** Child maltreatment, Parent-child agreement, Measurement

## Abstract

Parent-child agreement on child maltreatment was examined in a multigenerational study. Questionnaires on perpetrated and experienced child maltreatment were completed by 138 parent-child pairs. Multi-level analyses were conducted to explore whether parents and children agreed about levels of parent-to-child maltreatment (convergence), and to examine whether parents and children reported equal levels of child maltreatment (absolute differences). Direct and moderating effects of age and gender were examined as potential factors explaining differences between parent and child report. The associations between parent- and child-reported maltreatment were significant for all subtypes, but the strength of the associations was low to moderate. Moreover, children reported more parent-to-child neglect than parents did. Older participants reported more experienced maltreatment than younger participants, without evidence for differences in actual exposure. These findings support the value of multi-informant assessment of child maltreatment to improve accuracy, but also reveal the divergent perspectives of parents and children on child maltreatment.

Child maltreatment is a widespread phenomenon (Krug et al. 2002; Stoltenborgh et al. 2013). Child maltreatment is any act of commission (abuse) or omission (neglect) by a parent or other caregiver that results in actual or potential harm, or threat of harm to a child (Krug et al. 2002). Within this broad definition, five subtypes can be distinguished: sexual, physical, and emotional abuse, and physical and emotional neglect. Sexual abuse covers those acts where a parent or caregiver uses a child for sexual gratification. Physical abuse is defined as those acts of commission by a parent or caretaker that can cause actual physical harm (“non-incidental injury”). Emotional abuse is a chronic pattern of behaviors such as threatening, denigrating, humiliating, and ridiculing a child. Physical neglect refers to chronic failure of a parent or caretaker to provide a child with basic necessities such as food, clothing, shelter, medical care, educational opportunity, protection, and supervision. Emotional neglect is characterized by consistent failure of a parent or caretaker to provide a child with appropriate support, attention, and affection (Krug et al. 2002).

Estimates of the prevalence of child abuse vary considerably between studies. Meta-analyses have shown that self-reported prevalence is higher than prevalence reported by professionals (i.e., “sentinels”), such as general practitioners and well-baby clinic staff (Stoltenborgh et al. 2015). However, sentinel studies are fewer in number: the vast majority of studies use self- or parent report to estimate the prevalence of maltreatment (e.g., Briere and Elliot 2003; May-Chahal and Cawson 2005), which points to the need to examine similarities and differences between self-reported and parent reported parent-to-child maltreatment.

There is an ongoing debate about the accuracy and reliability of parental report on child maltreatment. Unfortunately, there is no gold standard to assess child maltreatment. While some research has suggested that parents can be reliable informants of child maltreatment (Kerker et al. 2000) and that they do report maltreatment committed against their own children (Straus et al. 1998), a number of studies suggest otherwise. For example, Lanyon et al. (1991) reported that 21% of parents suspected of involvement in child abuse completely deny, and 47% of parents partially deny allegations of abuse during an interview that was part of a CPS investigation. It is possible that parents underreport their committed maltreatment because of shame or fear of negative consequences of reporting. Since children are the victims, the use of child report on child maltreatment is sometimes considered to be more accurate (e.g., McGee et al. 1997; Winegar and Lipschitz 1999). However, children’s self-reports appear not to be free from bias either: underreporting has been documented (parent-child: Jouriles et al. 1997; sentinel-child: McGee et al. 1995; Widom and Morris 1997; Widom and Shephard 1996). In addition, self-report is not possible in the case of young children. Previous research thus suggests that neither parents nor children are reliable as single informants on child maltreatment, and has emphasized the need for a multi-informant approach to measuring child maltreatment (e.g., Brown et al. 1998; Chan 2015; Chan 2012; Kaufman et al. 1994; Kolko et al. 1996; McGee et al. 1995; Shaffer et al. 2008; Widom et al. 2015).

A crucial question that emerges in this context is whether parent report and child report on the prevalence and severity of maltreatment are convergent. Studies comparing parent and child report of parent-to-child maltreatment are scarce. In a study focusing on parent and adolescent report of parent-to-child maltreatment, Chan (2012) examined the agreement between parent-child matched pairs. Low to moderate agreement was found between parents and children (preceding year: kappa coefficients ranging from .28 to .34; lifetime: kappa coefficients ranging from .28 to .40). Moreover, children were more likely than their parents to report (very) severe physical violence. Parents, however, were more likely to report emotional abuse, neglect and minor physical assault. Similar levels of agreement were found in a recent study on the reliability of parental report of child victimization, comparing parent and adolescent reports of child maltreatment *by any adult* (Chan 2015). Kappa coefficients of agreement ranged from .23 (preceding year) to .29 (lifetime), indicating low agreement between parent and adolescent reports. Moreover, children were more likely than their parents to report child maltreatment. Similarly, Kolko et al. (1996) found that 6- to 13-year-old children reported considerably higher rates of mother-to-child violence than mothers did. In contrast, in a study comparing retrospective parent report and adolescent report of early childhood physical abuse, adolescents were found to report *fewer* physical abuse incidents than their parents (Tajima et al. 2004). In an earlier study, 7- to 9-year-old children were found to report lower levels of mother-child and father-child physical aggression than their mothers of fathers reported (Jouriles et al. 1997). Similarly, Kruttschnitt and Dornfeld (1992) reported that 11- to 12-year-old children were less likely than their mothers to disclose acts of maternal verbal aggression and violence in a sample of mothers who had been physically abused themselves. Finally, Jouriles and Norwood (1995) found that 4- to 14-year-old girls reported lower levels of maternal aggression towards them than their mothers reported.

How can we explain these differences between parental and child report of maltreatment? Previous research has put forward numerous factors affecting the reliability of parental report on parent-to-child maltreatment, such as fear of legal consequences, shame, denial (Appel and Holden 1998; Chan 2015; McGee et al. 1995), social desirability, minimizing of family problems (Milner and Crouch 1997), normativity (McGee et al. 1995; Straus et al. 1998), and recall bias due to the increased time between the maltreatment event and the report (Greenhoot 2011). Children’s report on parent-to-child maltreatment, on the other hand, may be influenced by embarrassment, the desire to protect parents out of a sense of loyalty, the wish to forget the victimization (Della Femina et al. 1990), the perception of having deserved punishment (Brown et al. 1998), or recall bias (Greenhoot 2011).

So far, studies comparing parent and child report of maltreatment found no significant correspondence between parents and children. However, most of these studies relied on dichotomized (yes/no) measures of maltreatment. Only two out of seven studies (Chan 2015; Chan 2012) examined both abuse and neglect. Two studies focused on both physical and emotional abuse (Kolko et al. 1996; Kruttschnitt and Dornfeld 1992), while the remaining three examined physical abuse only (Jouriles et al. 1997; Jouriles and Norwood 1995; Tajima et al. 2004). Moreover, child informants were confined to either younger children (range: 4–14 years) (Jouriles et al. 1997; Jouriles and Norwood 1995; Kolko et al. 1996; Kruttschnitt and Dornfeld 1992) or adolescents (Chan 2015; Chan 2012; Tajima et al. 2004) and three out of seven studies relied on mothers’ reports of maltreatment only and did not include father-to-child maltreatment (Jouriles and Norwood 1995; Kruttschnitt and Dornfeld 1992; Tajima et al. 2004). To address these limitations, in the present study we used continuous data of (incidence of parent-to-child maltreatment, we included children under and over 18 years of age reporting on their childhoods, and we included fathers as well as mothers.

## Research Questions and Hypotheses

The aim of the current study is to examine to what extent parents and children agree on the occurrence of various types of parent-to-child maltreatment. Specifically, we intended to (a) explore the *convergence* between parent- and child-reported incidence of maltreatment; and (b) investigate whether parents and children report *equal incidence* of child maltreatment (i.e., test for differences in mean scores between parents and children). Furthermore, (c) we tested effects of child age and gender on reports of maltreatment and on the agreement between parents and children.

Based on earlier research, we hypothesized that there would be no or low convergence between parents and children on the occurrence of child maltreatment. With regard to the reported incidence of maltreatment, previous studies were inconclusive on whether children or parents were more likely to report lower incidence of parent-to-child maltreatment.

## Method

### Sample

The current sample is a subsample from an ongoing, larger three-generation study on the intergenerational transmission of maltreatment. For this three-generation study, participants were recruited from two other studies that involved participants with an increased risk of parent-to-child maltreatment (Joosen et al. 2013, and Penninx et al. 2008). From these studies, we identified individuals who had at least one child of 8 years or older, and who had agreed to being invited for participation in future studies. We invited them to participate in the current study and, after their consent, invited their family members (parents, partners, children, siblings, nephews, nieces) to participate as well. This sampling strategy resulted in oversampling of individuals who reported that they had been maltreated in their childhood. The current study includes participants from the three-generation study who had been enrolled by 1 April, 2015.

To examine parent-child agreement on the occurrence of parent-to-child maltreatment, we selected pairs of participants from all three generations who reported on perpetrated (parents) and experienced (children) maltreatment. For reasons of clarity, we will hereafter use the term ‘children’ to refer to participants who report on childhood experiences. In the current paper, a considerable number of ‘children’ (75%) were (young) adults.

#### Families

The total sample consisted of 55 different families: 15 families with one child, 38 families with two children, and two families with three children. In total, 138 parent-child pairs participated. Out of 174 participants, 18 (10%) reported both as parent and as child.

#### Parents

Data were available for 83 parents (35 males, 48 females). The average age was 57.4 years (*SD =* 11.5, age range: 33–88 years). The vast majority of parents were Caucasian (95%), 3% were of Turkish descent, and 1% was of Latin-American descent. One participant was of unknown ethnicity. Eight percent of parents had completed elementary school, 21% had completed a short track of secondary school, 35% held an advanced secondary school or vocational school diploma, 22% held a college or university degree and 11% held a postgraduate diploma. Educational level of 3% of parents was unknown.

#### Children

Data for 91 children (33 males, 58 females) were included. The average age was 29.7 years (*SD =* 13.0, age range: 12–65 years). The vast majority of the children (91%) were Caucasian, 5% were Turkish, and 1% was of Latin-American descent. Three participants were of unknown ethnicity. Twenty-four percent of children were still in primary or high school. A small percentage of children (11%) had completed elementary school or a short track of secondary school, 34% had an advanced secondary school or vocational school diploma, 22% held a college or university degree and 6% held a postgraduate diploma. Education of 3% of children was unknown.

### Procedure

One or two family visits to the research lab were scheduled, depending on family composition. Adult participants visited the lab once with their core family – with their partner and child(ren), and once with their family of origin – with their sibling(s) and parent(s). Questionnaires on child maltreatment were completed during the first visit. Since all parents completed at least two of these questionnaires (one on experienced and another on perpetrated maltreatment), these questionnaires were scheduled as far apart as possible.

### Measures

#### Maltreatment

The Conflict Tactics Scales (CTS-PC; Straus et al. 1998) was administered to parents and children. Parents filled out the version that assessed the extent to which they had conducted specific physically or psychologically aggressive behaviors towards (each of) their child(ren). Children filled out the version that assessed to which extent they had experienced these behaviors from their father and/or mother.

The CTS-PC consists of 22 items comprising four scales, of which we excluded the *Nonviolent Discipline* scale (4 items), as it includes no items on maltreatment. *Psychological Aggression*, consisting of 5 items, assesses verbal or other non-physical communication aimed at inflicting psychological pain or fear to the child (e.g. “threatened to spank or hit”). Cronbach’s alphas for Psychological Aggression were adequate to good in the current sample (parent report: α_mother_ = .75, α_father_ = .64; child report: α_mother_ = .82, α_father_ = .77). *Physical Assault* consists of 13 items, including corporal punishment (5 items), severe assault (4 items), and very severe assault (4 items). Cronbach’s alphas for Physical Assault were adequate to good in the current sample (parent report: α_mother_ = .68, α_father_ = .71; child report: α_mother_ = .92, α_father_ = .93).

The *Neglect* scale (5 items) measures the failure of a parent to “engage in behavior that is necessary to meet the developmental needs of a child, such as not providing adequate food or supervision” (Straus et al. 1998, p. 253). We aimed to distinguish physical from emotional neglect in our analyses. Cronbach’s alphas for the four items about physical neglect were not sufficient (parent report: α_mother_ = .19, α_father_ = .48; child report: α_mother_ = .72, α_father_ = .67). Consequently, this scale was not included in analyses on different subtypes of maltreatment. Since the CTS-PC *Neglect* scale includes only one item on emotional neglect (failure to show or tell your child you love them), we added the five items of the *Emotional Neglect* scale from the Childhood Trauma Questionnaire (CTQ-SF; Bernstein et al. 2003). As a consequence, the *Emotional Neglect* scale consisted of six items (the five CTQ-items plus the one item on emotional neglect from the CTS-PC). Cronbach’s alphas for Emotional Neglect were adequate to excellent (parent report: α_mother_ = .79, α_father_ = .78; child report: α_mother_ = .94, α_father_ = .92).

In addition to the three subscale scores, we computed a Total maltreatment score, based on all subscales (including physical neglect). Cronbach’s alphas for this scale were adequate to excellent (parent report: α_mother_ = .82, α_father_ = .74; child report: α_mother_ = .94, α_father_ = .93).

To match the response categories of the CTS-PC and CTQ, we used a 5-point scale ranging from “never” (1) to “(almost) always” (5) for all items. For children 18 years or older, lifetime maltreatment (until 18 years) was assessed. For children who were under 18 years of age, parents and children reported on past-year maltreatment and maltreatment that occurred before the past year. The highest value for each item was used to calculate a scale score representing lifetime maltreatment.

### Analytical Strategy

To examine the absolute agreement between parents and children, the intraclass correlation coefficient was calculated (ICC (3,*k*), single measures, absolute agreement, see Shrout and Fleiss 1979) for the total maltreatment score and for each subtype of maltreatment. ICC (3,*k*) was employed with maltreatment of each “target” (i.e., the child) being rated by each of the two “judges” (i.e. parent, child), who were the only judges of interest.

Two sets of analyses were conducted to examine a) whether parents and their children agreed about the occurrence of different types of maltreatment, and b) if they reported similar levels of maltreatment: one set focusing on the association between parent-reported occurrence of maltreatment and child-reported occurrence of maltreatment (controlling for parent and child gender, and parent and child age), and another set focusing on the difference in means between parent- and child-reported occurrence of maltreatment.

#### Convergence between Parent- and Child-Reported Occurrence of Maltreatment

We used multilevel modeling with parent-reported incidence of maltreatment as a predictor and child-reported incidence of maltreatment as the outcome. We opted for a three-level design nesting parents (level 1) within children (level 2) within families (level 3). Because, within a family, parents share the same children, we considered parents repeated measures within children. Parent gender (level 1), child gender and child age (level 2) were added to the models as (fixed) covariates. Because parent age and child age were highly correlated (*r* = .92*, p* < .001) we only included child age. Since parent and child gender effects were not significant in any of the models, they were left out of the final analyses. Each model was run separately for the total maltreatment score and the three different types of maltreatment (emotional abuse, physical abuse, and emotional neglect).

First, we investigated the main effect of parent-reported maltreatment on child-reported maltreatment. We started by specifying an unconditional random intercept model with child-reported incidence of maltreatment as the outcome, to assess the intra-class correlation. In the first model, we included parent-reported incidence of maltreatment as a predictor. For the second model, we repeated the analyses including child age as a (fixed) covariate. The −2 log likelihood (−2LL) test indicates whether the new model is a better fit than the previous one. The size of this effect can be expressed in terms of the proportional reduction in variance (*PRV*) on the different levels of the model (Raudenbush and Bryk 2002; Singer and Willett 2003; Peugh 2010). This *PRV* is calculated as the difference between the variance of the model without the predictor of interest and the variance of the model that includes that predictor, divided by the variance of the model without the predictor ([*var*_NoPredictor_ - *var*_Predictor_]/ *var*_NoPredictor_).

Second, we examined moderating effects of parent gender, child gender, and child age on the relation between parent-reported and child-reported maltreatment by carrying out three analyses including interactions between parent-reported incidence of maltreatment and either 1) parent gender, 2) child gender, or 3) child age. In the moderator analysis focusing on parent gender, child gender was added as a covariate to control for the effect of child gender. Predictors were centered for all models. All models were tested with Full Maximum Likelihood estimation to enable comparison of nested models by inspecting the difference in deviances (−2 log likelihood). This difference is tested using the *Χ* ^2^-test with *df* equal to the difference in the number of estimated parameters between the models. We regard *p*-values smaller than .05 as indicating a significant difference in fit. As common in the multilevel context, we report unstandardized *β* weights.

One outlier was detected for child-reported physical abuse and, consequently, child-reported total maltreatment. These values were winsorized to values corresponding a standardized value of 3.29, while preserving the observed data ordering. Because the distributions of scores were skewed, both the parent and child report data were logarithmically transformed. The log-transformed data were then multiplied by 10 to scale up the variance.

#### Difference between Parent- and Child-Reported Incidence of Maltreatment

In the second set of analyses, we examined the difference in means between parent- and child-reported incidence of maltreatment. The data were restructured to accommodate one outcome measure per scale (e.g., *Psychological Aggression* reported by child and *Psychological Aggression* reported by parent were placed below each other). A variable to indicate the respondent (parent or child) was added. A four-level model predicting incidence of maltreatment was then formulated with respondent (level 1) nested within parent (level 2) nested within child (level 3), nested within family (level 4). Respondent was added to the model as a fixed factor. Testing this factor equals a paired samples *t*-test, corrected for the nested structure of the data.

Finally, as a sensitivity analysis, we repeated the analyses with the 10% participants who reported both as parent (potential perpetrator) and as child (about their childhood experiences) included only once. We randomly included them as either parent or child.

## Results

### Incidence of Maltreatment

Table 1 lists descriptive statistics on the incidence rates of reported maltreatment in the current study.

**Table 1:** Incidence rates of reported maltreatment

	Parent-report				Child-report			
	*M*	*SD*	Min.	Max.	*M*	*SD*	Min.	Max.
Emotional neglect (6–30)	10.18	3.19	6.0	20.0	11.91	4.97	6.0	30.0
Emotional abuse (5–25)	8.12	2.53	5.0	18.0	8.24	3.05	5.0	20.0
Physical abuse (13–65)	14.78	1.66	12.9	21.0	14.77	2.60	13.0	26.0
Total maltreatment (28–140)	37.32	5.06	28.0	53.0	39.24	9.06	28.0	83.0

NB Values between brackets indicate lowest through highest possible scores for each subscale

### Agreement between Parents and Children

Intraclass correlations were computed for father-child and mother-child pairs separately. It must be noted that intraclass correlations do not take into account the multilevel structure of the data. The agreement between parents and children was quite low (ICCs ≤ .40). The lowest levels of agreement were found for emotional neglect (see Table 2).

**Table 2:** Parent-child agreement on level of maltreatment (ICC)

	ICC(3,*k*)	
	Father-child	Mother-child
Total maltreatment	.29	.18
Emotional abuse	.36	.40
Physical abuse	.28	.31
Emotional neglect	.13	.18

### Relation between Parent- and Child Report: Multilevel Analyses

#### Total Maltreatment

With total maltreatment as the outcome, the intraclass correlations (ICC) indicated that differences between children accounted for 39% of the variance in child-reported incidence of maltreatment (*p* = .001), and that 24% of the variance was accounted for by differences between families (*p* = .056). The remaining 37% was residual variance between parents (see Table 3). These values indicate that a considerable part of the variance was accounted for by the nested structure of the data. Parent-reported incidence of maltreatment was included as a predictor in Model 1, and child age was added in Model 2 (see Table 4). Differences between -2LL values revealed that including parental report as a predictor (*β* = 0.27, *t* = 2.44, *p* = .016) significantly improved the model, *Χ* ^2^ (1) = 5.45, *p* = .020. The *PRV* for parent-reported total maltreatment on level 1 (parent level) was −0.02, so level 1 residual variance stayed virtually the same after adding parental report. The *PRV* for parent-reported maltreatment on level 2 (child level) equaled 0.18, so 18% of the intercept variance at level 2 was explained by including parent report as a predictor. Level 3 (family level) intercept variance increased by 8% (*PRV* = −0.08). This indicates that adding parental report as a predictor eliminated some of the variance at the child level (see Table 3).

**Table 3:** Variance accounted for (ICC) and proportional reduction in variance (PRV) on parent, child and family level

	Total maltreatment		Emotional abuse		Physical abuse		Emotional neglect	
	ICC^a^	PRV^b^	ICC^a^	PRV^b^	ICC^a^	PRV^b^	ICC^a^	PRV^b^
Level 1: parent	.37	-0.02	.40	-0.02	.63	0.03	.24	<−0.01
Level 2: child	.39**	0.18	.34**	0.21	.05	0.57	.59**	0.03
Level 3: family	.24	-0.08	.26*	0.12	.32**	0.07	.17	0.03

* p < .05; ** p < .01

^a^ICC for baseline model

^b^PRV for model 1

**Table 4:** Main multilevel models of child-reported maltreatment (total score) predicted by parent-reported maltreatment (total score)

Model		Baseline model	Model 1 Parent report ^a^	Model 2 Parent report and age child ^b^
Fixed effects				
Initial status	Intercept	15.88 (.10)**	15.88 (.10)**	15.87 (.09)**
	Age child			0.03 (.01)**
	Parent report		0.27 (.11)*	0.27 (.11)*
Variance Components				
Level 1	Father	0.01 (.06)	0.04 (.06)	0.07 (.06)
	Mother	0.35 (.09)**	0.33 (.09)**	0.31 (.08)**
Level 2	Child	0.39 (.12)**	0.32 (.11)**	0.29 (.10)**
Level 3	Family	0.24 (.12)	0.26 (.12)*	0.15 (.09)
Goodness-of-fit				
Deviance		312.27	306.82	291.31
Parameters		5	6	7
∆ χ^2^			5.45 (1)*	15.51 (1)**

*p < .05; **p < .01

^a^ Compared to baseline model

^b^ Compared to model 1

Controlling for child age again improved the model significantly (*β* = 0.03, *t* = 4.21, *p* < .001), *Χ* ^2^ (1) = 15.51, *p* < .001. Older children tended to report more maltreatment than younger children did. Altogether, the predicted association between parent-reported and child-reported occurrence of parent-to-child total maltreatment was significant, even after controlling for child age.

To examine whether similar effects would be found for the different subtypes of maltreatment, additional analyses were conducted for these subtypes.

#### Emotional Abuse

Findings were parallel to the overall model with total maltreatment (see Tables 3 and 5 for more detail). Again, the nesting effect was considerable. Differences between -2LL values revealed that including parental report as a predictor (*β* = 0.28, *t* = 3.56, *p* < .001) significantly improved the model, *Χ* ^2^ (1) = 11.58, *p* < .001. *PRV*s for parent-reported emotional abuse are listed in Table 3. *PRV*s indicated that adding parental report as a predictor mostly eliminated variance at the child level. Controlling for child age (*β* = 0.03, *t* = 2.25, *p* = .029) improved the model even further, *Χ* ^2^ (1) = 4.80, *p* = .028. Again, older children were more inclined to report emotional abuse than younger children.

**Table 5:** Main multilevel models of child-reported emotional abuse predicted by parent-reported emotional abuse

Model		Baseline model	Model 1 Parent report ^a^	Model 2 Parent report and age child ^b^
Fixed effects				
Initial status	Intercept	8.88 (.17)**	8.89 (.16)**	8.88 (.15)**
	Age child			0.03 (.01)*
	Parent report		0.28 (.08)**	0.30 (.08)**
Variance Components				
Level 1	Father	0.21 (.18)	0.29 (.18)	0.33 (.18)
	Mother	0.86 (.25)**	0.82 (.23)**	0.79 (.23)**
Level 2	Child	0.91 (.29)**	0.71 (.25)**	0.68 (.25)**
Level 3	Family	0.70 (.34)*	0.62 (.29)*	0.52 (.28)
Goodness-of-fit				
Deviance		452.71	441.13	436.33
Parameters		5	6	7
∆ χ^2^			11.58 (1)**	4.80 (1)*

*p < .05; **p < .01

^a^ Compared to baseline model

^b^ Compared to model 1

#### Physical Abuse

Variances accounted for on the different levels of the baseline model are listed in Table 3. Findings paralleled those of total and emotional abuse (see Tables 3 and 6 for more detail). Differences between -2LL values revealed that including parental report as a predictor (*β* = 0.34, *t* = 3.47, *p* < .001) significantly improved the model, *Χ* ^2^ (1) = 11.31, *p* < .001). The *PRV* for parent-reported physical abuse on level 1 (parent level) was 0.03, indicating that residual variance decreased by 3%. *PRV*s for parent-reported physical abuse indicated that adding parental report as a predictor mostly eliminated variance at the child level (see Table 3). Controlling for child age (*β* = 0.02, *t* = 3.29, *p* = .002) again improved the model significantly, *Χ* ^2^ (1) = 9.50, *p* = .002. Older children reported more physical abuse than younger children.

**Table 6:** Main multilevel models of child-reported physical abuse predicted by parent-reported physical abuse

Model		Baseline model	Model 1 Parent report ^a^	Model 2 Parent report and age child ^b^
Fixed effects				
Initial status	Intercept	11.60 (.07)**	11.60 (.07)**	11.60 (.06)**
	Age child			0.02 (<.01)**
	Parent report		0.34 (.10)**	0.32 (.10)**
Variance Components				
Level 1	Father	0.05 (.04)	0.07 (.03)	0.08 (.04)*
	Mother	0.32 (.07)**	0.29 (.06)**	0.28 (.06)**
Level 2	Child	0.03 (.04)	0.01 (.03)	0.01 (.03)
Level 3	Family	0.19 (.06)**	0.18 (.05)**	0.14 (.04)**
Goodness-of-fit				
Deviance		228.72	217.41	207.911
Parameters		5	6	7
∆ χ^2^			11.31 (1)**	9.50 (1)**

*p < .05; **p < .01

^a^ Compared to baseline model

^b^ Compared to model 1

#### Emotional Neglect

The ICCs with emotional neglect as outcome are reported in Table 3. Parent report of emotional neglect (Model 1), and parent report of emotional neglect corrected for child age (Model 2) were entered into the subsequent models (see Table 7). Differences between -2LL values revealed that including parental report (*β* = 0.12, *t* = 1.56, *p* = .123) as a predictor did not significantly improve the model, *Χ* ^2^ (1) = 2.37, *p* = .124. *PRV*s for parent-reported emotional neglect showed that adding parental report as a predictor eliminated some of the variance at the child level (see Table 3). Controlling for child age (*β* = 0.05, *t* = 3.48, *p* = .001) improved the model, *Χ* ^2^ (1) = 10.96, *p* < .001. Again, older children reported more emotional neglect than younger children did. The predicted association between parent- and child-reported occurrence of parent-to-child emotional neglect was significant after controlling for child age.

**Table 7:** Main multilevel models of child-reported emotional neglect predicted by parent-reported emotional neglect

Model		Baseline model	Model 1 Parent report ^a^	Model 2 Parent report and age child ^b^
Fixed effects				
Initial status	Intercept	10.59 (.19)**	10.59 (.19)**	10.56 (.17)**
	Age child			0.05 (.01)**
	Parent report		0.12 (.08)	0.13 (.08)
Variance Components				
Level 1	Father	0.18 (.21)	0.21 (.21)	0.26 (.20)
	Mother	0.62 (.25)**	0.59 (.24)*	0.54 (.23)*
Level 2	Child	1.94 (.53)**	1.87 (.52)**	1.83 (.49)**
Level 3	Family	0.54 (.49)	0.53 (.52)	0.23 (.39)
Goodness-of-fit				
Deviance		472.40	470.03	459.07
Parameters		5	6	7
∆ χ^2^			2.37 (1)	10.96 (1)**

*p < .05; **p < .01

^a^ Compared to baseline model

^b^ Compared to model 1

### Parent Gender, Child Gender, and Child Age

Next, the main and interactive effects of parent and child gender and child age were tested. No main effects of parent and child gender were found, indicating no differences between boys and girls nor fathers and mothers. In addition, no significant moderating effects of parent and child gender were found (*ps* > .145), indicating that the relation between parent- and child-reported incidence of maltreatment did not depend on parent and child gender.

Last, the moderating role of child age on the relation between parent- and child-reported maltreatment was examined. A significant interaction between child age and parent-reported incidence of emotional abuse was found (*β* = −0.01, *t* = −2.13, *p* = .036), indicating a moderating role of child age in the association between child-reported and parent-reported incidence of parent-to-child emotional abuse. For younger children, the association between parent- and child-reported emotional abuse was stronger than for older children. No moderating effects of child age were found for total maltreatment, physical abuse, or emotional neglect (*ps* > .639).

### Differences between Parent- and Child Reports

A significant difference between parents and children was found for total maltreatment (*t* = −2.16, *p* = .032), indicating that children reported significantly more parent-to-child maltreatment than parents (*M*_child_ = 39.35, *SD*_child_ = 9.62; *M*_parent_ = 37.32, *SD*_parent_ = 5.06). The same was true for emotional neglect: Children reported significantly more parent-to-child emotional neglect than parents did (*t* = −3.78, *p* < .001) (*M*_child_ = 11.91, *SD*_child_ = 4.97; *M*_parent_ = 10.18, *SD*_parent_ = 3.19). Note that taking the variances on child and family level into account, absolute differences were small. There were no differences between parent- and child-reported incidence of emotional abuse and physical abuse (*p*s > .541). Running the analyses on the log-transformed data did not alter the results.

### Sensitivity Analysis and Exposure Effect

Results did not alter when the 10% participants who reported both as parent and as child were randomly included as either parent or child. To explore whether the main effect of age was in fact an exposure effect (i.e. that older children report more maltreatment because they could have been exposed to maltreatment for a longer period of time), we reran the first set of analyses excluding children under 18 years (*n* = 23) because the exposure time only varied for these participants; the participants who were 18 years and older all had 18 years of exposure time. Results remained virtually unchanged: differences between betas ranged from .01 (emotional abuse) to .04 (total maltreatment). Thus, the age effect was robust, with older child participants reporting higher levels of maltreatment, and this effect was not due to duration of exposure.

## Discussion

Using a parent-child matched sample, the present study found that: (a) agreement between parents and children was quite low, especially for emotional neglect, (b) there was a positive but weak relation between parent and child report for all subtypes of parent-to-child maltreatment, (c) older children reported more maltreatment than younger children, (d) parents and children reported similar incidence of emotional and physical abuse, but children reported slightly more parent-to-child total maltreatment – particularly emotional neglect – than parents did, (e) younger children converged more with their parents on reported emotional abuse than older children, and (f) no effects were found for parent or child gender. We had expected limited convergence between parents and children on reported child maltreatment. Although higher parental report of maltreatment tended to be accompanied by higher child report (i.e. there was convergence between parents and children), our results point to modest associations between parents and children with regard to the level of (all subtypes of) maltreatment. These results are in line with earlier studies (e.g., Chan 2015; Kolko et al. 1996; Tajima et al. 2004).

With respect to absolute differences between parent- and child-reported incidence of parent-to-child maltreatment, we found that parents and children on average reported an equal level of emotional and physical abuse. However, in comparison to their children, parents on average reported somewhat less emotional neglect and, as a result, slightly lower total maltreatment (a total score comprised of all subtypes of abuse, including physical neglect). Indeed, parent-child agreement was lowest for emotional neglect and total maltreatment. Parents reporting less maltreatment than their children is not uncommon in maltreatment literature (e.g. Chan 2015; Kolko et al. 1996). One possible explanation is that neglect is a less tangible subject, since it encompasses acts of *omission* (i.e. the absence of certain behaviors), which may make it more difficult for parents to report: it might be easier for children to indicate what they have missed than it is for parents to indicate what they did not do. In addition, five out of six questions concerning emotional neglect were positively formulated (e.g. “My father/mother made me feel loved”). There might be a gap between what parents feel toward their child and what they convey: parents may have felt love for their children but may have been unable to sufficiently convey this to their child. These discrepancies are less likely to appear when it concerns the rating of more observable behaviors, such as hitting or screaming at a child. As an alternative or additional explanation, parents may simply not consider specific events or situations as evidence for neglect that their children *do* consider as evidence of neglect. Following this line of reasoning, our results might also point to a generation gap, and reflect changing attitudes and beliefs about appropriate parenting practices.

Older children reported more maltreatment than younger children. It seems reasonable to assume that children over 18 years of age report more maltreatment than younger children simply because they have been exposed to it longer than younger children (exposure effect). However, excluding the younger children from the sample resulted in similar findings, refuting the explanation of an exposure effect. Therefore we believe that these results mirror the gradual shift in social values; some parenting disciplines are simply not socially acceptable anymore. This would support the view that public education efforts calling attention to corporal punishment and physical abuse have had an effect on societal attitudes towards maltreatment (Straus and Gelles 1986). What used to constitute normal discipline (e.g. spanking) might now amount to excessive discipline or maltreatment. Furthermore, differences in reminiscence, the process of recalling personal experiences from the past, could also play a role. Studies have shown that an overrepresentation of memories from the adolescent and early adult years is present in the autobiographical memories of older adults (e.g., Conway and Pleydell-Pearce 2000; Fitzgerald 1988). Older children in our study (that is, children in their role as respondent, but clearly in the adult age range) may therefore have reported more maltreatment than younger children because these memories are better accessible to them, especially considering the saliency of the events (Cowan and Davidson 1984). On the other hand, younger children might be hesitant to report maltreatment because of fear of consequences.

The finding that younger children converged more than older children with their parents on the occurrence of emotional abuse might point to differences in attribution between younger and older child informants. Older child informants may report progressively more emotional abuse, because they have come to realize that their parents’ behavior was not normative. However, the interaction effect was small, indicating that it should be interpreted with caution.

A limitation of the current study that should be noted is the retrospective nature of the reports. Although the degree of retrospect varies – some participants report on more recent experiences than others – recall bias might have affected the accuracy of these reports. Events may have been selectively recalled, or even forgotten, and memories may be subject to distortion (e.g., McGee et al. 1995; Quas et al. 1997). A prospective longitudinal design (e.g., Tajima et al. 2004), with data collection at the time that the event occurs, reduces the likelihood of forgotten memories or memories that are distorted by later experiences. Prospective longitudinal studies on child maltreatment, however, face the challenge that recent maltreatment might be underreported because of respondents’ fear of legal consequences.

The current study, although modest in sample size, is one of the few multi-informant studies on parent-to-child maltreatment comparing children’s and parents’ reports. We examined parent-to-child maltreatment in a high-risk sample, with the advantage that in a substantial number of families maltreatment incidents *have* occurred, which enables the comparison of parent and child report without having to deal with distributions that are too skewed to warrant this type of analysis (because the majority of parent-child dyads would converge on the absence of maltreatment). Using continuous instead of dichotomous measures provides a more precise estimate of the occurrence of parent-to-child maltreatment. Moreover, our study included both abuse and neglect, whereas the vast majority of prior studies have focused exclusively at child abuse. A final strength of our study is that it included both mothers *and* fathers, and younger *and* older children. The results are therefore likely to represent a rather comprehensive approach.

Our results indicate that parents and children provide unique perspectives on parent-to-child maltreatment. Moreover, our findings emphasize the need of a multi-informant approach to measuring maltreatment to improve accuracy: involving additional informants who are neither perpetrator nor victim may add to the accuracy of the assessment of maltreatment.
